# CD8^+^ tumor‐infiltrating lymphocytes within the primary tumor of patients with synchronous *de novo* metastatic colorectal carcinoma do not track with survival

**DOI:** 10.1002/cti2.1155

**Published:** 2020-07-17

**Authors:** Rosemary Millen, Shona Hendry, Vignesh Narasimhan, Rebecca Abbott, Matthew Croxford, Peter Gibbs, Jeanne Tie, Hui‐Li Wong, Ian Jones, Suzanne Kosmider, David Byrne, John Zalcberg, Stephen Fox, Jayesh Desai, Kumar Visvanathan, Robert G Ramsay, Ben Tran

**Affiliations:** ^1^ Peter MacCallum Cancer Centre Melbourne VIC Australia; ^2^ Sir Peter MacCallum Department of Oncology University of Melbourne Melbourne VIC Australia; ^3^ St. Vincent's Hospital Melbourne VIC Australia; ^4^ Department of Anatomical Pathology St Vincent's Hospital Melbourne Melbourne VIC Australia; ^5^ Department of Pathology University of Melbourne Melbourne VIC Australia; ^6^ Western Health Footscray VIC Australia; ^7^ Royal Melbourne Hospital Melbourne VIC Australia; ^8^ Walter and Eliza Hall Institute Parkville VIC Australia; ^9^ Monash University Melbourne VIC Australia; ^10^ Alfred Health Prahran VIC Australia; ^11^ University of Melbourne Melbourne VIC Australia

**Keywords:** advanced metastatic colorectal cancer, PD‐L1, tumor infiltrating lymphocytes

## Abstract

**Objectives:**

Tumor‐infiltrating lymphocytes (TIL), particularly CD8^+^ TILs in patients with colorectal cancer (CRC), are highly prognostic in the early‐disease stages (I‐II). In metastatic disease (stage IV; mCRC), their influence is less well defined. It has presumably failed to contain tumor cells to the primary site; however, is this evident? We explored the prognostic impact of TILs at the primary site in patients who presented *de novo* with mCRC.

**Methods:**

Treatment‐naïve patients (109) with mCRC were assessed for CD8^+^ TILs and PD‐L1 expression. Microsatellite instability (MSI) was evaluated by IHC for PMS2 and MSH6 proteins and/or by PCR using the Bethesda panel.

**Results:**

Microsatellite instability‐high tumors had significantly more CD8^+^ TILs, with no significant survival advantage observed between MSI‐H and microsatellite stable (MSS) tumors (12 vs 19 months, *P* = 0.304). TIL density for all cases had no impact on OS (low: 20 vs high: 13 months, *P* = 0.426), while PD‐L1 of 1% or higher was associated with reduced mean survival (9.6 vs 18.9 months; *P* = 0.038). MSI‐H tumors and associated immune cells had higher PD‐L1 expression than in MSS cases. A positive correlation between PD‐L1 on immune cells and CD8+ve TILs was found. A subset of MSS tumors had relatively high TILs approximating that of MSI‐H tumors.

**Conclusion:**

In contrast to early‐stage CRC, the immune response in primary tumors of patients with *de novo* mCRC does not appear to influence survival. A subgroup of MSS tumors was identified with increased TILs/PD‐L1 comparable to MSI‐H tumors, traditionally not be considered for immune checkpoint blockade and perhaps should be.

## Graphical abstract

Unlike early‐stage colorectal cancer (CRC), the immune response in primary tumors of patients with *de novo* mCRC does not appear to influence survival despite MSI‐H tumors having higher tumor infiltrating lymphocytes (TILs). This unique cohort of patients with synchronous mCRC by definition represents the breakdown of immune containment of the primary tumor. Despite this, we also identified a subgroup of MSS tumors with a high TILs comparable to MSI‐H tumors and this group traditionally would not be considered for immune checkpoint blockade, and perhaps should be.

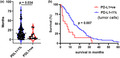

## Introduction

The majority of CRC‐related mortality is a result of metastatic disease, when metastatic disease is detected at initial presentation/diagnosis (*de novo* metastatic) or when the primary tumor has been treated with curative intent, and subsequently, distant/metastatic recurrence develops.^1^ Approximately 15–25% of patients present with metastatic disease at the time of diagnosis,^2^ and stage IV CRC (mCRC) has a 5‐year survival of 12–14%.^3^ Therefore, there is a need to investigate these tumors in more detail and explore new therapeutic options. In the current age of immunooncology, there has been substantial interest in better understanding the tumoral immune response in this disease, which is largely resistant to such approaches.

In CRC, a favorable tumor‐infiltrating lymphocytes (TILs) appears to be associated with improved survival independent of TNM staging.^4^ The Immunoscore^®^ developed by Galon and Pages *et al*.^4^ has found a strong association between increased CD8^+^ infiltrate and favorable prognosis in primary CRC. These studies under‐represent stage IV mCRC, and therefore, other studies have investigated the prognostic significance of the Immunoscore in patients with mCRC. Xie *et al*.^5^ evaluated TILs in a cohort of mCRC patients and found a high Immunoscore was an independent prognostic marker and a lower Immunoscore was significantly associated with synchronous disease. Rozek *et al*.^6^ reported on a cohort CRC patients including 69 stage IV patients and found a higher Immunoscore was associated with an improved disease‐specific survival (DSS). This cohort also evaluated patients with MSI‐H tumors and found the Immunoscore was prognostic for improved survival in patients with MSI‐H tumors, in line with the current dogma; however, there was no evaluation in the context of stage IV MSI‐H tumors.

Despite immunotherapy being relatively ineffective in the majority of mCRC, a small subset of patients with microsatellite unstable tumors (MSI‐H) can gain benefit.^7^ Patients with MSI‐H stage I‐III CRC tumors have an improved prognosis compared to patients with microsatellite stable (MSS) tumors, whereas patients with stage IV MSI‐H tumors have a worse outcome than patients with stage IV MSS mCRC.^8^, ^9^ The consensus molecular subtype was introduced in 2015 as a way to classify CRC tumors into four subtypes. Subtype CMS1 identifies hypermutable characteristics, MSI‐H along with CIMP^+^ phenotype with BRAF frequently mutated, low somatic copy number alteration, immune infiltration and worse prognosis. By contrast, there was no MSS molecular subtype that includes immune infiltrate.^10^

The use of immune‐checkpoint blockade (ICB) has demonstrated impressive activity in MSI‐H mCRC. The CheckMate‐142 study showed durable responses following treatment with nivolumab, an anti‐PD‐1 antibody, alone and in combination with ipilimumab, an anti‐CTLA‐4 antibody, in a subgroup of stage IV CRC patients with MSI‐H tumors.^7^ The KEYNOTE‐164 study showed durable responses following treatment with pembrolizumab, another anti‐PD‐1 antibody, in patients with mCRC that were MSI‐H.^11^ The KEYNOTE‐177 trial is currently exploring the role of first‐line therapy in a phase III study in patients with mCRC with MSI‐H tumors.^12^ Despite the promise around immune ICB in MSI‐H mCRC, the failure rate of 50–60% in MSI‐H tumors leaves substantial room for improvement.^13^

In patients with mCRC with MSS tumors, there has been a void of ICB activity. However, a recent randomised phase II study showed a modest survival benefit from the combination of durvalumab (anti‐PD‐L1) and tremelimumab (anti‐CTLA‐4) in patients with advanced treatment‐refractory CRC compared to best supportive care, suggesting that a subset of patients with MSS mCRC may benefit from treatment with ICB.^14^

Clearly, a robust predictive biomarker is required to identify the 30% of patients with MSI‐H tumors and the smaller proportion of patients with MSS tumors who may benefit from ICB. Despite some limitations, the expression of PD‐L1 by immunohistochemistry (IHC) is advanced as a predictive biomarker for anti‐PD‐1 therapy in non‐small‐cell lung cancer and some other tumor types, and continues to be explored in the context of MSI tumors.^15^ Given the relationship between MSI‐H and elevated cytotoxic CD8^+^ T‐cell infiltrate,^16^ it is of interest to explore PD‐L1 expression in *de novo* mCRC.

In this study, we investigated the immune response in a unique cohort of patients with metastatic disease CRC at diagnosis, herein defined patients presenting with *de novo* mCRC. We explored the role of microsatellite status on the immune response and aimed to determine whether there was a subset of MSS tumors that harbor an immune response that approximates that of MSI‐H tumors. Such insights may inform the development of biomarkers for ICB in both MSI and MSS mCRC.

## Results

### Patient and tumor characteristics

In all, 109 patients with *de novo* mCRC who underwent primary tumor resection (Table 1) and had available archival tumor specimens were included in this study, and IHC analysis was performed on 104 tumor specimens. The median OS in the entire cohort was 19 months. Microsatellite status was defined as either deficient MMR (dMMR)/MSI‐H (*n* = 12, 11%) or proficient MMR (pMMR)/MSS (*n* = 97, 89%), as outlined in Table 1. In univariate hazard ratio analysis, we found that patients who were 65 years and older had a significantly reduced OS as seen in Table 2. No other factors affected survival in this cohort of patients, including microsatellite status (detailed below).

**Table 1:** Patient and tumor characteristics

Clinical characteristics	Overall	dMMR/MSI‐H	MSS
Total, *n*	109	12	97
Age (years), median	28–89, 89	48–87, 74	28–89, 69
Female, *n* (%)	50 (46)	8 (67)	42 (43)
Male, *n* (%)	59 (54)	4 (33)	55 (57)
Pathology tumor stage			
T2, *n* (%)	2 (2)	1 (8)	1 (1)
T3, *n* (%)	48 (44)	5 (42)	43 (44)
T4, *n* (%)	59 (54)	6 (50)	53 (55)
Colon, *n* (%)	99 (91)	12 (100)	87 (90)
Rectum, *n* (%)	11 (10)	0 (0)	10 (10)
Right‐sided	45 (41)	10 (83)	46 (47)
Left‐sided	59 (54)	1 (8)	57 (59)
Unknown sidedness	5 (5)	1 (8)	4 (4)

**Table 2:** Univariate and hazard ratios

	Univariate HR (95% CI)	*P*‐value
Age group		
Up to 65		
> 65	1.61 (1.12–2.30)	0.01*
Gender		
Female		
Male	0.84 (0.60–1.17)	0.293
T stage		
T2		
T3	0.38 (0.12–1.24)	0.108
T4	0.75 (0.24–2.40)	0.631
Site of original		
Colon		
Rectum	0.76 (0.42–1.37)	0.362
MSI		
pMMR		
dMMR	1.37 (0.74–2.51)	0.315
CD8^+^ per mm^2^		
Low		
High	1.18 (0.78–1.79)	0.44
Tumor PD‐L1		
< 1%		
1% or more	1.42 (0.77–2.61)	0.265
MYB CT		
Low		
High	0.61 (0.27–1.39)	0.236
MYB IM		
Low		
High	0.90 (0.40–2.04)	0.803
GRP78 CT		
Low		
High	1.26 (0.79–2.03)	0.336
GRP78 IM		
Low		
High	1.12 (0.73–1.72)	0.604
RAD21		
Low		
High	0.65 (0.40–1.07)	0.09

Univariate analysis of patient and tumor characteristics; *p = 0.01 highlighting statistical significance.

### Overall patient survival with MSI‐H and MSS tumors

No significant difference in OS was seen between patients with MSI‐H/dMMR and MSS/pMMR tumors, as shown in Supplementary figure 1A and B [(median 12 months; 95% CI: 6.13–17.87) MSI/dMMR vs 19 months (95% CI 13.34–24.66) MSS/pMMR, log‐rank *P* = 0.304].

### CD8^+^ T‐cell differences between MSI‐H and MSS tumors

**Figure 1:**
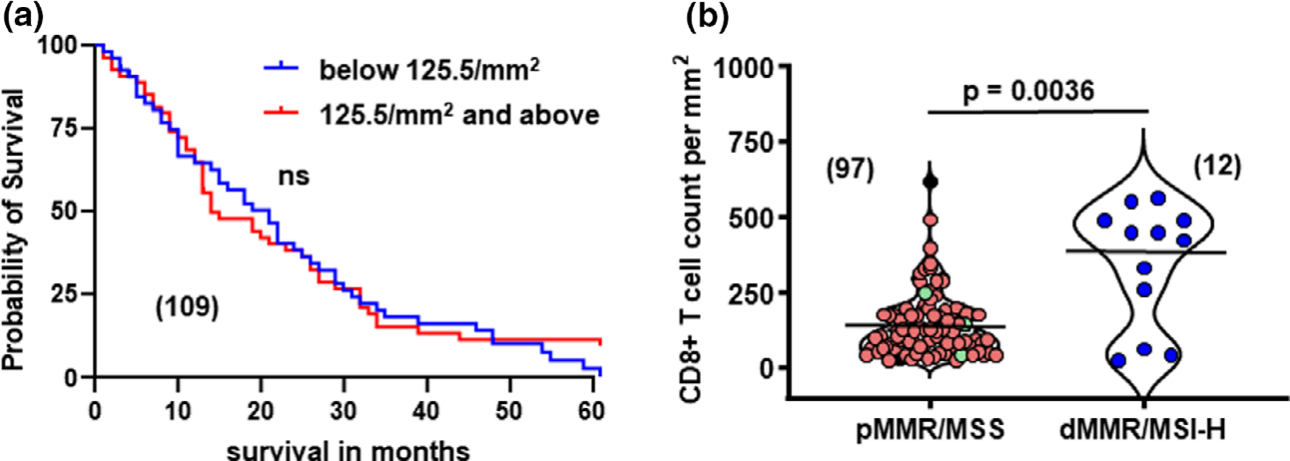
No overall survival advantage observed when cytotoxic CD8^+^ T‐cell infiltration is assessed in primary tumors of patients with *de novo* mCRC. **(a)** Kaplan–Meier survival curve showing OS stratified on CD8^+^ low (blue line) vs CD8^+^ high (green line) against months, log‐rank *P* = 0.700; *n* = 109, and includes dMMR/MSI‐H cases. **(b)** Violin plots showing overall CD8^+^ count per mm^2^ in tumors that are pMMR/MSS (red) and dMMR/MSI‐H (blue) in log scale. Green symbols in microsatellite stable group were selected as outliers for further microsatellite testing; dotted line shows median cut‐off for CD8^+^ infiltrate at 125.5 and 435.5 CD8^+^ T cells per mm^2^ for pMMR/MSS (*n* = 97) and dMMR/MSS (*n* = 12), respectively; two‐tailed unpaired *t*‐test, **P = 0.0036. Slides were scored by two independent pathologists.

The median CD8^+^ T‐cell density of 125.5 cells per mm^2^ was defined as the cut‐off for high and low TILs for the entire cohort, including both MSI‐H and MSS tumors. If MSI‐H/dMMR cases were excluded, the cut‐off became 123.5 cell per mm^2^ but by both measures (123.5 or 125.5 cells per mm^2^), there was no significant difference in patient survival (Figure 1a). Nevertheless, the CD8^+^ T‐cell infiltrate was significantly higher in MSI‐H tumors than MSS tumors (MSI‐H mean: 344.4 cells per mm^2^ vs MSS mean: 147.8 cells per mm^2^, *P* = 0.0036) (Figure 1b), outlined in Table 3. To further elaborate, a higher density of CD8^+^ TILs was not associated with improved OS (median 20 months for low CD8^+^ (95% CI 15.21–24.79) vs 13 months for high CD8^+^ (95% CI 8.07–17.93), log‐rank *P* = 0.426).

**Table 3:** Immune cell characteristics

Immune characteristics	MSI‐H	MSS	*P*‐value
Median CD8^+^ infiltrate per mm^2^	435.5 cells per mm^2^	123.5 cells per mm^2^	
Mean CD8^+^ infiltrate per mm^2^	344.4 cells per mm^2^	147.8 cells per mm^2^	0.0036^a^
%PD‐L1 expression TC (mean)	21%	0.7%	< 0.0001^a^
%PD‐L1 expression IC (mean)	3%	0.6%	0.0015^a^

A total of 104 patients.

^a^ Two‐tailed *t*‐test.

### PD‐L1 expression

**Figure 2:**
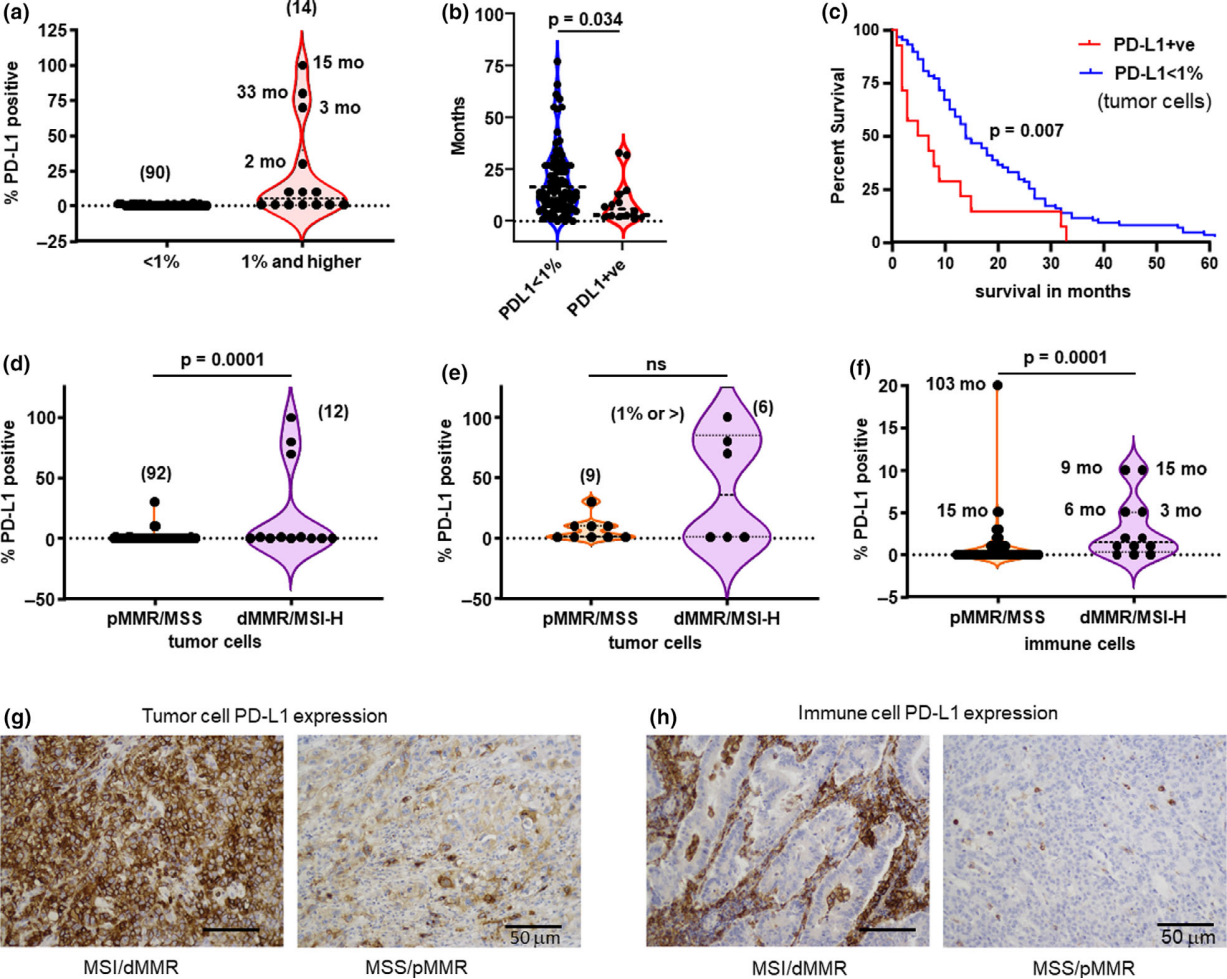
PD‐L1 tumor expression > 1% is found mostly on dMMR/MSI‐H tumors. **(a)** < 1% (*n* = 90) and 1% or higher (*n* = 14) PD‐L1 expression on tumor cells from the primary tumor of patients with *de novo* mCRC, *n* = 104. Associated survival of patients in months (mo) from whom the tumors were obtained in parentheses. **(b)** Violin plots showing months of survival in patients with tumors expressing < 1% (blue line) or > 1% PD‐L1 (red line) on tumor cell surface where OS is inferior in cases with PD‐L1 ≥ 1% two‐tailed unpaired *t*‐test, *P* = 0.034. **(c)** Kaplan–Meier survival curve showing OS stratified on tumor cells expressing < 1% (blue line) or ≥ 1% PD‐L1 (red line) on tumor cell surfaces [*P* = 0.007, log‐rank Mantel–Cox test]. **(d)** Violin plot of percentage of PD‐L1 expression on tumor cells in pMMR/MSS (red, *n* = 92) and dMMR/MSI‐H (blue, *n* = 12); two‐tailed unpaired *t*‐test, ns: *P* < 0.0001. **(e)** Violin plots showing percentage of ≥ 1% PD‐L1 expression on tumor cells in pMMR/MSS (red, *n* = 9) and dMMR/MSI‐H (blue, *n* = 6); two‐tailed unpaired *t*‐test, ns. **(f)** Violin plot of percentage of PD‐L1 expression on immune cells in pMMR/MSS (red, *n* = 92) and dMMR/MSI‐H (blue, *n* = 12); two‐tailed unpaired *t*‐test, *P* = 0.0001. Notable months (mo) of survival corresponding to high cases shown. **(g, h)** Representative images of low and high PD‐L1 expression on tumor and immune cells in each cohort including stromal and intraepithelial immune cells, scale bar = 50 μm. Slides were scored by two independent pathologists. Representative images of CD8^+^ infiltrate (brown stain) in two patients with either a MSS tumor (left panel) or a microsatellite unstable tumor (right panel), scale bar = 50 μm.

Generally, PD‐L1 expression was not detected across the majority of the samples assessed in the cohort; only 13% (14/103) of all cases showed PD‐L1 expression (Figure 2a). Nevertheless, to evaluate those cases with any expression, we selected PD‐L1 expression of 1% and higher on tumor cells (TC) as a cut‐off based on the previous PD‐L1 IHC assays in CRC^17^ to ask whether this minor subset of mCRC might have a survival benefit. Overall survival was worse in patients with TC expression of PD‐L1 ≥ 1% than < 1% (*P* = 0.034; two‐sided *t*‐test) (Figure 2b), and when depicted as a Kaplan–Meier plot, median OS for PD‐L1 < 1% on TC was 14 months compared to 6 months ≥ 1% (Mantel–Cox test, log‐rank *P* = 0.007) (Figure 2c). PD‐L1 expression was more frequent on tumor cells in dMMR/MSI‐H cases (Figure 2d) but when compared to pMMR/MMS cases with PD‐L1 expression of 1% or greater, both tumor types were indistinguishable (Figure 2e). Immune cell PD‐L1 expression was similarly greater in dMMR/MSI‐H cases (Figure 2f). Even so, there was no survival difference when considering PD‐L1 expression on ICs (Supplementary figure 2A) or when considering high and low PD‐L1 specifically on dMMR/MSI‐H tumors (Supplementary figure 2B). Representative IHC images showing the spectrum of PD‐L1 expression are shown in Figure 2g and h. These data suggest that in this group of patients, PD‐L1 expression on TC tracks with relatively poor survival.

### PD‐L1 expression and CD8^+^ infiltrate

**Figure 3:**
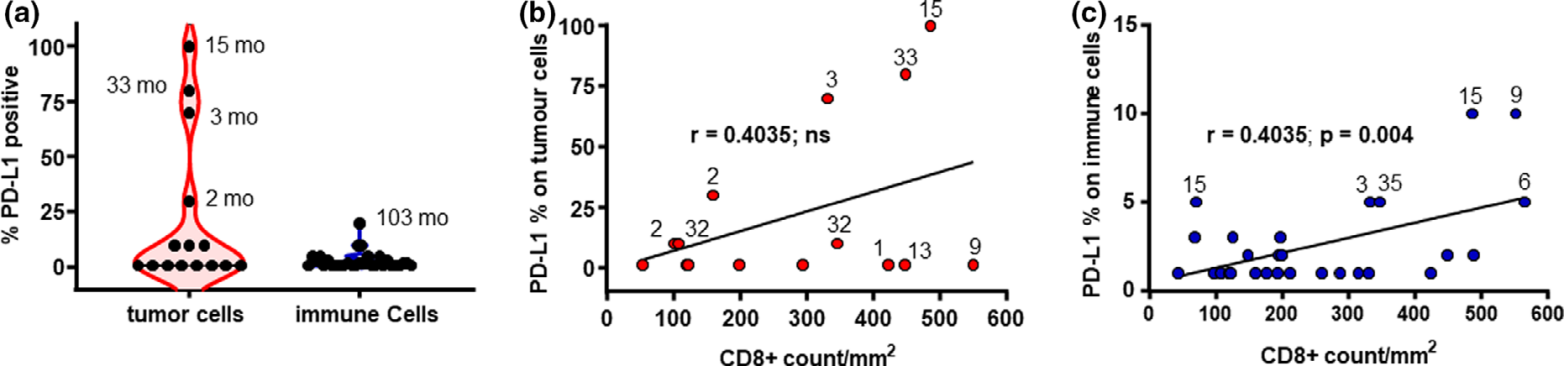
PD‐L1 immune cell expression associated with CD8^+^ T‐cell infiltration on tumor and immune cells. **(a)** Violin plots showing percentage of PD‐L1 positivity for tumor cells defined as a percentage of tumor cells (TC) with any discernible membrane staining (red) and immune cells (IC). **(b)** XY plots showing CD8^+^ count per mm^2^ in combined regions and PD‐L1 expression on tumor cells for those patients with positive PD‐L1 expression, *n* = 15, correlation analysis, *r* = 0.4035, ns and **(c)** immune cells for those patients with positive PD‐L1 expression, *n* = 31, two‐tailed correlation analysis, *r* = 0.5177, *P* = 0.004. Associated numbers correspond to months (mo) of patient survival.

Highlighted in Figure 3a is the range of expression of PD‐L1 on TC and IC. Individual survival in months is shown for the tumors with exceptional PD‐L1 expression. The three patients identified in Figure 2c with high PD‐L1 expression on TCs indeed had a high CD8^+^ T‐cell infiltrate (331, 449 and 486 mm^−2^) as shown in Figure 3b. Despite these three patients having a high T‐cell infiltrate, each patient had an OS of 3, 33 and 15 months, respectively, and overall, there was no correlation between PD‐L1 and CD8^+^ cell counts (Figure 3b). There was, however, a positive correlation between IC PD‐L1 expression and increased CD8^+^ T‐cell infiltrate (*P* = 0.004) (Figure 3c), suggesting that the presence of CD8+ve cells is perhaps influenced more so by PD‐L1 expression on the IC and not on the TC.

### Small MSS cohort that approximate MSI‐H

Despite MSS tumors overall having a lower infiltration of CD8^+^ T cells than MSI‐H, some MSS tumors (*n* = 4) had a high CD8^+^ T‐cell infiltrate, greater or equal to MSI‐H tumors (mean MSI‐H CD8^+^ infiltrate: 344 cells per mm^2^) as seen in Figure 1b. The MSS tumors with increased CD8+ve TILs (green symbols) were further investigated using the Bethesda PCR panel that interrogated five microsatellite loci,^18^ and all were confirmed as MSS.

## Discussion

Fifteen to twenty‐five per cent of patients with CRC will present with synchronous metastatic disease at the time of diagnosis^19^ and have a particularly poor survival, with 5‐year survival of 14%.^20^, ^21^, ^22^ While MSI‐H is typically a good prognostic factor in stage I‐III CRC, it is a poor prognostic factor in mCRC. This may, however, be confounded by higher proportions of *BRAF* mutations and right‐sided primaries in the MSI‐H group, both of which are also poor prognostic features.^23^ Although the activity of ICB in MSI‐H mCRC has been impressive, a substantive proportion of patients gain no benefit. Additionally, for MSS mCRC, only the smallest proportion of patients gain benefit, if any; however, patients with *POLE* mutations may be an exception.^24^ Therefore, we still await a biomarker that will identify which patients are best treated with ICB.

Here, we investigated a unique cohort of stage IV *de novo* mCRC patients with available archival tumor specimens through primary tumor resection and examined the relationship between CD8^+^, PD‐L1 and microsatellite status. As expected, we identified a higher density of CD8^+^ T cells in MSI‐H CRC vs MSS (median: MSI‐H: 435.5 mm^−2^ vs MSS: 123.5 mm^−2^). This observation alludes to why mCRC MSI‐H patients respond to ICB in the current clinical trials, as CD8^+^ T cells are present in higher numbers and some of these patients are able to be rescued by ICB. Xie *et al*. reported on a similar cohort evaluating TILs in resected primary colorectal tumors of patients with synchronous disease. This study included 302 samples and found that TILs were significantly associated with better OS.^5^ However, their cohort had a lower frequency of MSI‐H tumors than our cohort, and in our cohort, high CD8+ve TILs did not track with superior OS.

Unlike early‐stage CRC (I‐II), where high TILs are associated with a good prognosis and therefore improved OS compared to low TIL infiltrate tumors, in metastatic stage IV CRC, there is no difference in OS, which may exclude TILs being good in this setting. When we performed this analysis in the pMMR/MSS group alone, the same result was found (Supplementary figure 3). This is the antithesis of what is observed in stage I‐III CRC, with high TILs associated with improved survival.^4^ Thus, these observations highlight the different state of the immune response in the advanced setting compared to earlier stages of CRC and it may be likely that tumor control by the immune infiltrate has been lost.

A major finding from our study was identification of a small proportion of mCRC MSS tumors that did have a high infiltration of CD8^+^ T cells, *equivalent* to or greater than MSI‐H tumors. A recent study by Chen *et al*. is challenging the dogma that only mCRC MSI‐H tumors respond to ICB. In the CCTG CO.26 trial, the vast majority of patients treated had MSS tumors, two patients had dMMR, and 12 patients had unknown MMR status. The durvalumab (anti‐PD‐L1) and tremelimumab (anti‐CTLA‐4) combination provided an additional 2.5‐month median OS compared to best supportive care [median OS 6.6 vs 4.1 months (hazard ratio (HR) = 0.72; *P* = 0.07)]. Although these differences are small, this study demonstrates synergy of two immune checkpoint targets, which may be the preferred strategy when treating MSS mCRC. Our finding of a small subset of MSS mCRC with high CD8^+^ density may further explain the small survival benefits seen in this study. Similarly, a recent report by Fakih *et al*.^25^ characterised a risk group that accounted for 10% of CRC patients that had a high TIL infiltrate, but poor prognosis, irrespective of MSI status.

We investigated potential associations between PD‐L1 expression on TCs and ICs with microsatellite status and CD8^+^ T‐cell infiltrate. We quantified PD‐L1 expression on TC and used the cut‐off of 1% or higher, based on a previous study using this cut‐off as a predictive biomarker for response to anti‐PD‐L1 therapy in CRC.^17^ It is accepted that an inflammatory environment, namely secretion of IFN‐γ by infiltrating immune cells, within the tumor microenvironment (TME) can lead to up‐regulation of PD‐L1 on tumor cells.^26^ Although we did not find a significant relationship between TC PD‐L1 expression and infiltrating CD8^+^ T cells, we observed a significant association between increased IC PD‐L1 expression and infiltrating IC. Deciphering which types of immune cells are expressing PD‐L1 would be of interest, to determine whether these are indeed regulatory immune cells. It is accepted that myeloid‐derived suppressor cells (MDSCs) and T‐regulatory cells have the ability to express PD‐L1 and these cells can suppress the immune response at the TME in CRC.^27^ Nevertheless, PD‐L1 expression in < 1% of tumor cells had significantly better survival than those patients with tumor harboring 1% or greater PD‐L1 expression. This indicates that these primary tumors are likely to be immunologically suppressed but we can only speculate that this may the case at the metastatic site also.

This is a unique cohort of mCRC with available tissue from primary resected tumors at the time of metastatic disease. Although our cohort consisted of consecutive patients from a real‐world registry, all patients underwent primary tumor resection, which introduces an inherent selection bias for (generally) healthier patients, and thus, this cohort may not be representative of the entire population. A limitation of this study was not having access to the tissue blocks of matched metastatic tumors, which may have provided additional insight into the immune response at the distant metastatic site. Furthermore, assessment of PD‐1 expression on the infiltrating immune cells would have provided greater insight into the axis that may exist between PD‐L1 and PD‐1. Determining which TILs express PD‐L1 would also be beneficial in defining which subset of immunosuppressive cells are present in these tumors. Additionally, CD3 was not included as an immune marker as all CD8^+^ T cells are CD3^+^. Nevertheless, to remain consistent with the Immunoscore which uses both CD3^+^ and CD8^+^, this should have been included in our study. A final limitation was a small sample size. This may have resulted in a type II error because of low sample size, which could explain why we could not demonstrate a survival advantage.

Future studies should therefore compare synchronous resected primary and metastatic tumors for immune differences including CD8^+^ T cells, PD‐L1 expression and other immune checkpoint expression. Through integrative analysis including genetic evidence of immunoediting, other studies found the immune component in CRC to be more superior to MSI status alone.^28^ Measuring other immune signatures including infiltrate context and organisation and molecular signatures to determine effector function should be considered.^25^ Furthermore, interrogating the genomic landscape of synchronous primary and metastatic tumors in ICB trials may provide insight into why only some patients respond to ICB.

Despite these above limitations, our study demonstrates that in MSI‐H CRC, while CD8^+^ density is significantly higher, the proportion of CD8^+^ with corresponding PD‐L1‐positive tumors is only 20%. This important finding may explain the relatively low response rates to ICB (compared to other tumor types such as melanoma) but that identifying such cases may direct ICB more effectively. Additionally, in the MSS cohort, we were able to identify a small subset of tumors with very high CD8^+^ density approximating that seen in the MSI‐H cohort. This finding might explain the results from CO.26 study and/or have identified a small subset of MSS patients who may gain benefit from ICB. By understanding the immune response in mCRC in more depth, this will help advance possible immunotherapies for these patients.

## Methods

### Population

This is a retrospective analysis of a prospectively maintained data cohort. This cohort of patients has been described in detail previously.^29^ Briefly, consecutive patients from the Royal Melbourne Hospital and The Western Hospital, Melbourne, who underwent primary tumor resection for treatment‐naïve *de novo* mCRC between 1 January 2000 and 1 July 2010 were investigated. Patients were excluded if primary tumor resection had occurred after any systemic therapy or if they had, at any time, undergone resection of metastatic disease with curative intent.

### Ethics

Ethical approval was obtained from the Melbourne Health Human Ethics Committee, HREC2011.225.

### Data collection

Baseline characteristics collected included age, gender, co‐morbidities, MSI status, date of diagnosis, date of surgery, pathology tumor stage, side of original tumor and previous chemotherapy treatments. Overall survival was defined as date of surgery to date of death or was censored at date of last follow‐up. Cox univariate hazard ratio model analyses were performed to identify factors affecting overall survival (OS).

### Immunohistochemistry‐automated staining

FFPE blocks were cut at 3 μm thickness and transferred onto Superfrost Plus slides (Thermo Fisher Scientific, Waltham, MA, USA). These were stained on a Ventana Benchmark Ultra auto‐stainer (Ventana Medical Systems Inc, AZ, USA). Antibodies used were as follows: anti‐CD8^+^ clone 4B11 (NCL‐CD8‐4B11, Leica Biosystems, Wetzlar, Germany) at 1:100 dilution, anti‐PDL1 SP263 (790‐4905, Ventana Medical Systems Inc) neat, anti‐PMS2 EPR3947 (288R‐18‐ADR, Cell Marque, Rocklin, CA USA) neat and anti‐MSH6 44 (08‐1374, Invitrogen, Pleasanton, CA, USA) at 1:800. FFPE human placenta, tonsil, lymph node, normal colon and CRC were used as controls.

### Evaluation of immunohistochemistry

Immunohistochemical evaluation of mismatch repair (MMR) protein expression is established as an equivalent alternative method of identifying MMR‐deficient tumors to PCR‐based microsatellite testing, with similar sensitivity between 2‐protein and 4‐protein panels.^30^, ^31^ Deficient mismatch repair (dMMR) was defined as loss of either PMS2 or MSH6 in tumor nuclei, with appropriate staining in adjacent normal tissue. Confirmation and validation of high microsatellite instability (MSI‐H) and microsatellite stability (MSS) was determined by using the Bethesda PCR panel that interrogated five microsatellite loci.

The invasive margin (IM) was defined as a 1‐mm‐wide region centred on the border separating the benign tissue from malignant glands, and central tumor (CT) was defined as the remainder of the tumor. Slides were scored by two blinded and independent investigators (RM and SH).

A pathologist selected two 0.56 mm^2^ ‘hot spots’, defined as 740 × 540 pixels at 1.181 mm per pixel, per region (CT and IM) for analysis. Tumor‐infiltrating lymphocytes were defined as intraepithelial and intrastromal cells within the tumor tissue. CD8^+^ T cells were defined as positive CD8 receptor expression with immune cell morphology. CD8^+^ T cells were quantified as positive cells per mm^2^ in combined regions of central tumor (CT) and invasive margin (IM), following the guidelines of the ‘Immunoscore^®^’ by scoring two ‘hot‐spot’ areas in each region, and averaging the counts.^32^ CD8^+^ infiltrates were classified as ‘high’ or ‘low’, compared to the median count per mm^2^. PD‐L1 positivity for tumor cells (TC) was defined as percentage of tumor cells with any discernible membrane staining, and for infiltrating immune cells (IC) as the percentage of tumor area covered by PD‐L1‐positive immune cells. A cut‐off value of 1% was set for TC PD‐L1 expression. Areas of surface ulceration, necrosis, macrophages and neutrophils associated with necrotic areas, and normal lamina propria in any non‐invasive component were excluded.

### Statistical analyses

All data analysis was performed using IBM^®^ SPSS^®^ version 22 and GraphPad 7.0d. IBM Armonk, New York, USA; GraphPad San Diego, CA USA. Continuous parametric and non‐parametric variables were expressed as means with standard deviation and medians with ranges, respectively. Categorical variables were expressed as numbers with percentages. Patients were stratified into two groups based on MSI status. Kaplan–Meier survival analysis was performed to evaluate OS. Overall survival was calculated from time of surgery to last known date of follow‐up (censored data), or date of death. The Log‐rank test was used to assess statistical significance. Factors affecting survival were identified using univariate analysis. A *P*‐value < 0.05 was considered to be statistically significant.

## Conflict of interest

The authors declare no conflict of interest.

## Author contributions

**Rosemary Millen:** Conceptualization; Data curation; Formal analysis; Investigation; Methodology; Writing‐original draft; Writing‐review & editing. **Shona Hendry:** Data curation; Formal analysis; Investigation; Methodology; Writing‐original draft; Writing‐review & editing. **Vignesh Narasimhan:** Data curation; Formal analysis; Methodology; Writing‐original draft; Writing‐review & editing. **Rebecca Abbott:** Data curation; Investigation; Methodology; Writing‐review & editing. **Matthew Croxford:** Data curation; Investigation; Resources; Writing‐review & editing. **Peter Gibbs:** Conceptualization; Investigation; Resources; Writing‐review & editing. **Jeanne Tie:** Investigation; Resources; Writing‐review & editing. **Hui‐Li Wong:** Data curation; Investigation; Resources; Writing‐review & editing. **Ian Jones:** Investigation; Resources; Supervision; Writing‐review & editing. **Suzanne Kosmider:** Data curation; Resources; Writing‐review & editing. **David Byrne:** Data curation; Formal analysis; Investigation; Methodology; Writing‐original draft; Writing‐review & editing. **John Zalcberg:** Conceptualization; Funding acquisition; Resources; Supervision; Writing‐review & editing. **Stephen Fox:** Conceptualization; Data curation; Formal analysis; Funding acquisition; Investigation; Methodology; Project administration; Resources; Supervision; Writing‐original draft; Writing‐review & editing. **Jayesh Desai:** Investigation; Project administration; Resources; Supervision; Writing‐review & editing. **Kumar Visvanathan:** Conceptualization; Funding acquisition; Investigation; Methodology; Project administration; Resources; Supervision; Writing‐original draft. **Robert G Ramsay:** Conceptualization; Data curation; Formal analysis; Funding acquisition; Investigation; Methodology; Project administration; Resources; Supervision; Writing‐original draft; Writing‐review & editing. **Ben Tran:** Conceptualization; Data curation; Formal analysis; Funding acquisition; Investigation; Methodology; Project administration; Resources; Writing‐original draft; Writing‐review & editing.
