# Supplementary figures and images for: CD8+ tumor‐infiltrating lymphocytes within the primary tumor of patients with synchronous de novo metastatic colorectal carcinoma do not track with survival
Source: Clin Transl Immunology. 2020 Jul 17;9(7):e1155. doi: 10.1002/cti2.1155 (PMC7484874; doi:10.1002/cti2.1155)

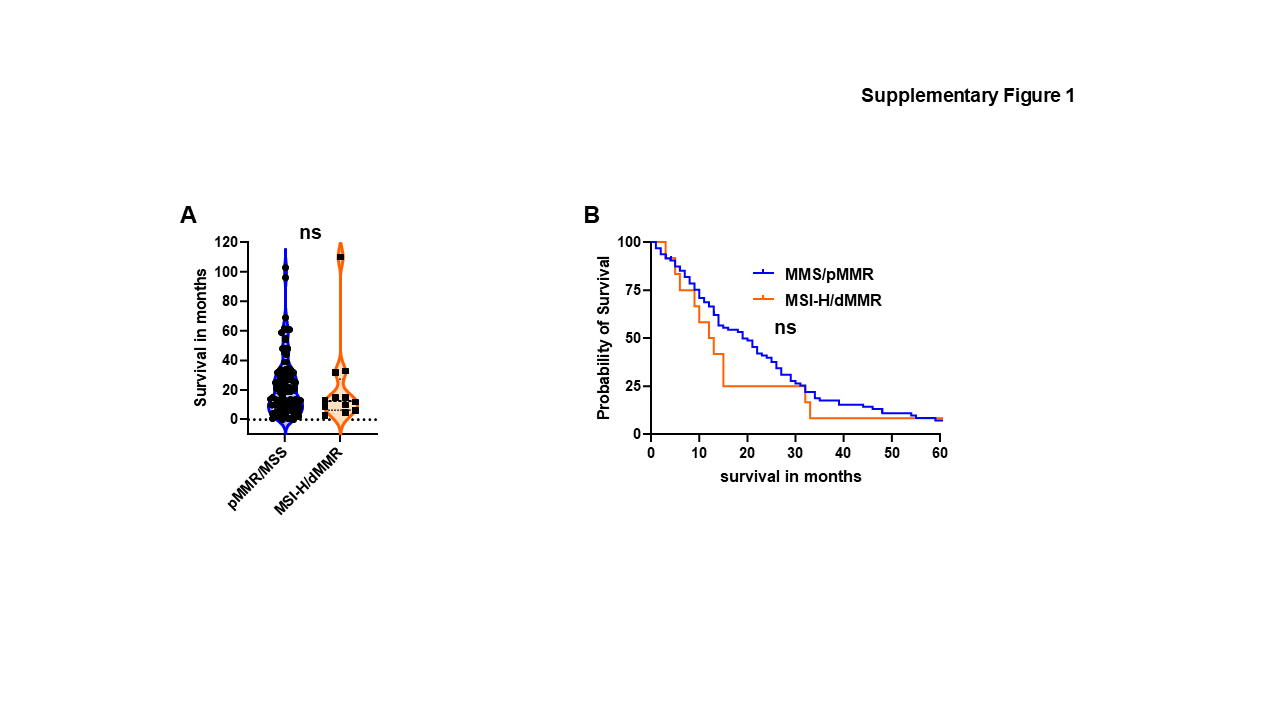

Supplement: Supplementary file 1 [file CTI2-9-e1155-s001.tif]

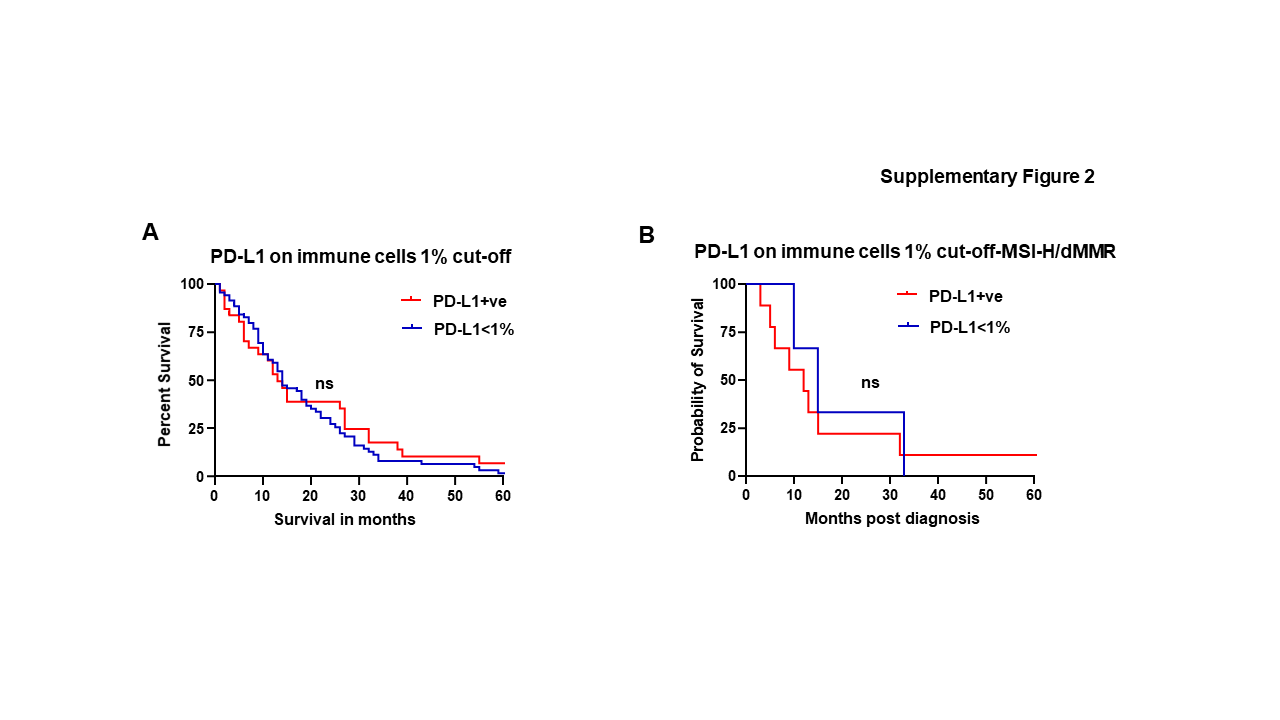

Supplement: Supplementary file 2 [file CTI2-9-e1155-s002.tif]

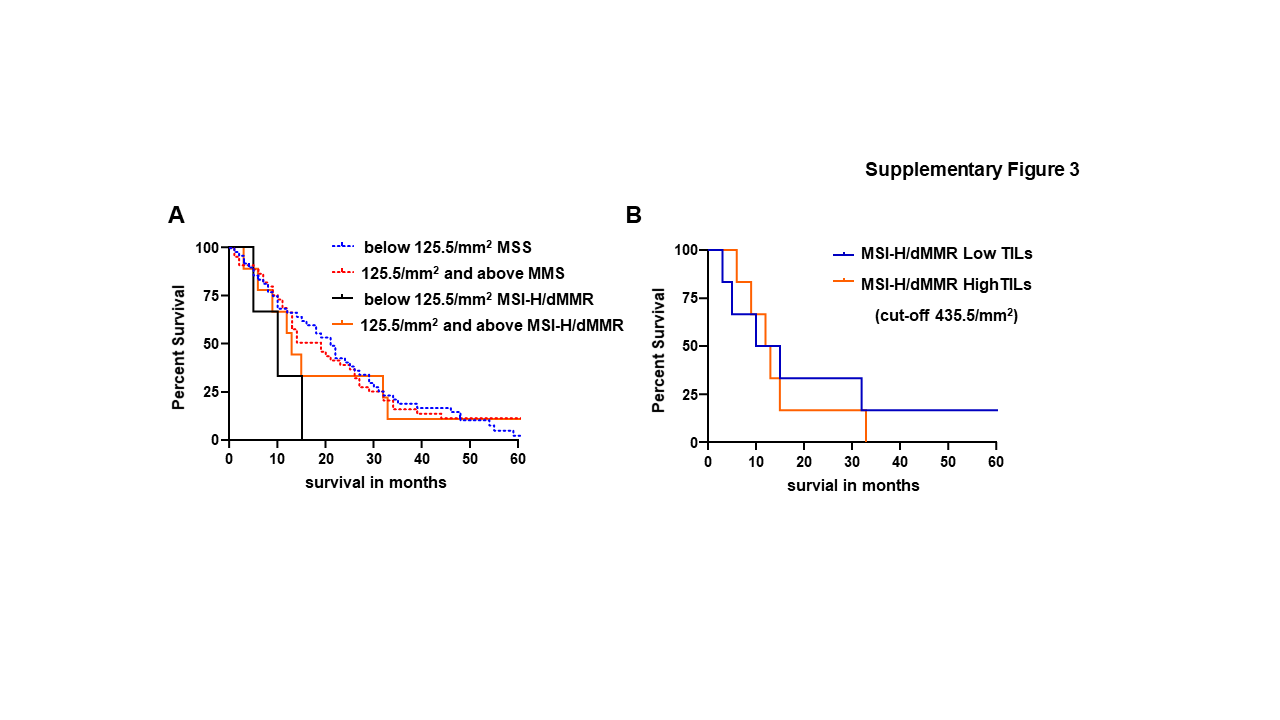

Supplement: Supplementary file 3 [file CTI2-9-e1155-s003.tif]
