# Supplementary material for: CD8+ tumor‐infiltrating lymphocytes within the primary tumor of patients with synchronous de novo metastatic colorectal carcinoma do not track with survival
Source: Clin Transl Immunology. 2020 Jul 17;9(7):e1155. doi: 10.1002/cti2.1155 (PMC7484874; doi:10.1002/cti2.1155)
Supplement: Supplementary file 4 [file CTI2-9-e1155-s004.docx]

**Supplementary Figure 1. Mismatch repair deficiency is not associated with a survival advantage in *de novo* mCRC tumors**. **(A)** Violin-plots showing months survival based upon MSI-status. **(B)** Kaplan-Meier survival curve showing OS stratified on microsatellite status of patients with *de novo* mCRC. Patients were stratified as MSI-H/dMMR (red line) vs. MSS/pMMR (blue line) against months. Median survival was 12 months (MSI-H/dMMR) and 19 months (MSS/pMMR), n=116 patients in total, MSI-H/dMMR n=12, MSS/pMMR n=97. Median survival for dMMR 19 months compared to 12 months for pMMR (log rank p=0.704).

**Supplementary Figure 2. No survival advantage is observed in patients when tumors assessed for PD-L1 expression on immune cells**. **(A)** Kaplan-Meier survival curve showing OS against months, stratified on PD-L1 ≥1% (red) and >1% PD-LI on immune cells (blue). Median survival 22 months for low vs. 13 months for high (log rank p = 0.38). **(B)** Kaplan-Meier survival curve showing OS against months, stratified on PD-L1 ≥1% (red) and >1% PD-LI on immune cells (blue) in MSI/dMMR cases. Median survival 15 months for low vs. 12 months for high (log rank p = 0.60).

**Supplementary Figure 3. No survival advantage is observed in patients with pMMR tumors when assessing CD8**^+^ **infiltrate**. **(A)** Kaplan-Meier survival curve showing OS against months, stratified on CD8^+^ infiltrate as CD8^+^ low (blue) and CD8^+^ high (red) based upon cut-off for total cohort (median - 125.5 cells/mm^2^). **(B)** Kaplan-Meier survival curve showing OS against months, stratified on CD8^+^ infiltrate as CD8^+^ low (blue) and CD8^+^ high (red) based upon cut-off for MSI-H/dMMR cohort (median - 435.5 cells/mm^2^).
